# RTMB: An R Package for Fitting Latent Variable Models With Template Model Builder

**DOI:** 10.1002/ece3.74572

**Published:** 2026-10-29

**Authors:** Kasper Kristensen, Anders Nielsen, Christoffer Moesgaard Albertsen, Casper Willestofte Berg, Vanessa Trijoulet

**Affiliations:** ^1^ National Institute of Aquatic Resources Technical University of Denmark Lyngby Denmark

**Keywords:** automatic differentiation, ecology, epidemiology, fisheries science, hierarchical models, Laplace approximation, state‐space models, TMB

## Abstract

Hierarchical and latent variable models are fundamental in ecology, fisheries science, and epidemiology, but fitting them efficiently relies on automatic differentiation (AD) combined with approximations such as the Laplace approximation. Template Model Builder (TMB) has become a standard tool for this kind of inference in R, but it requires users to write the objective function in C++, which is a potential obstacle for those unfamiliar with it, slows model iteration through compilation, and complicates debugging and simulation. We present RTMB, an R package that exposes the full power of TMB's AD machinery directly from plain R. Users write the joint negative log‐likelihood as an ordinary R function; RTMB traces it onto an AD tape and hands the result to TMB's existing optimization, Laplace approximation, and reporting routines. We describe the design of the package, illustrate its use through a worked example, and provide a side‐by‐side comparison with the equivalent TMB implementation of a theta‐logistic state‐space population model. The result is a transparent, debuggable, and pedagogically accessible interface to state‐of‐the‐art latent variable inference, with runtime performance comparable to native TMB.

## Graphical abstract

Hierarchical and latent variable models are fundamental in ecology, fisheries science, and epidemiology, but fitting them efficiently relies on automatic differentiation (AD) combined with approximations such as the Laplace approximation. RTMB is an R package that exposes the full power of TMB's AD machinery directly from plain R. Users write the joint negative log‐likelihood as an ordinary R function. The result is a transparent, debuggable, and pedagogically accessible interface to state‐of‐the‐art latent variable inference, with runtime performance comparable to native TMB.

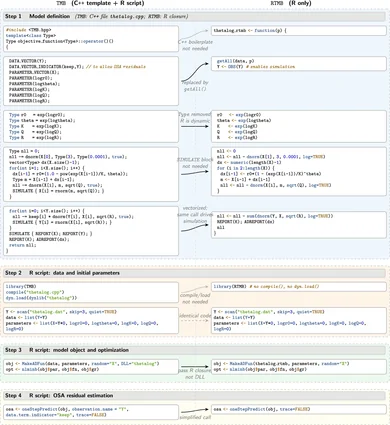

## 1 Introduction

Many central quantities in ecology, such as population size, recruitment, movement, and survival, are latent and must be inferred from observed data through hierarchical models that are often analytically intractable. Efficient inference for such models typically requires likelihood gradients, which are supplied exactly and at scale by automatic differentiation (AD). Building on ADMB (Fournier et al. 2012) and ADMB‐RE (Skaug and Fournier 2006), TMB (Kristensen et al. 2016) combined sparse Hessian computation with the Laplace approximation, making models with thousands of random effects routinely tractable.

Through TMB, ecologists and statisticians routinely fit models that would otherwise be intractable: zero‐inflated mixed models in glmmTMB (Brooks et al. 2017), spatiotemporal models such as sdmTMB (Anderson et al. 2025) and VAST (Thorson 2019), state‐space stock assessments such as SAM (Nielsen and Berg 2014) and WHAM (Stock and Miller 2021), multispecies models (Trijoulet et al. 2019; Trijoulet et al. 2020; Thorson et al. 2024), and animal‐movement models (Albertsen et al. 2015).

Despite its widespread adoption, TMB requires users to write the objective function in C++, imposing a steep learning curve for R‐proficient users. Each model change triggers a slow edit‐compile‐debug cycle, with error messages that are often opaque. Simulation adds further complexity: every density call must be paired with a manual SIMULATE {…} block that duplicates the model logic.

The RTMB package (Kristensen 2023) addresses these challenges by providing a native R interface to TMB's AD machinery. The central function is MakeADFun(), which takes a user‐defined objective function (the joint negative log‐likelihood) and constructs a model object that TMB can optimize and apply the Laplace approximation to. RTMB's version of MakeADFun() accepts an R closure directly as the objective function: every arithmetic operation is recorded onto an AD tape (a computational trace used to compute exact derivatives automatically), which is then passed to TMB's existing optimization and Laplace approximation routines. No C++ code is written or compiled.

Once MakeADFun() has been invoked, all downstream TMB functionality works exactly as before and at comparable speed. This design gives RTMB several compelling advantages: (i) models can be changed and re‐run instantly with no compilation step, (ii) standard R debugging tools work inside the objective function, (iii) simulation and one‐step‐ahead (OSA) residuals (Thygesen et al. 2017; Trijoulet et al. 2023) are handled automatically and coding is simplified, (iv) automatic consistency checks of the model in simulation mode are performed, returning informative errors when inconsistencies are detected (e.g., if the negative log‐likelihood is accumulated more than once, or if a stochastic variable lacks a corresponding simulation statement or is simulated multiple times), (v) external R packages can be called inside the objective function, and (vi) the entire workflow is accessible without C++ experience.

This manuscript describes the design and implementation of RTMB, illustrates its use through a worked example, and provides a direct comparison with equivalent TMB code. Our aim is to demonstrate that RTMB lowers the barrier to building and fitting latent variable models in R, without sacrificing the numerical performance or flexibility that established TMB as a standard tool in ecology, fisheries science, epidemiology, and many other fields.

## 2 Technical Description of RTMB

This section describes how RTMB works under the hood. Readers who are primarily interested in seeing the package in action can skip directly to Section 3, which walks through a complete worked example and return here for reference as needed.

### 2.1 Package Architecture

In practical terms, RTMB accepts a statistical model written as a plain R function and passes it to TMB's numerical machinery, which handles the heavy lifting of computing derivatives and approximating integrals over random effects. No C++ code is written or compiled. Under the hood, RTMB couples R directly to TMB's TMBad automatic‐differentiation engine (a replacement for the older CppAD backend used in standard TMB, Bell (2005)): the C++ interface handles the low‐level AD machinery, while the substantial R‐side implementation provides distribution overloading (the same density call behaves differently depending on whether the model is being fit, simulated from, or checked), automatic simulation triggering, OSA residuals, and memory‐safe tape management.

The package is organized into conceptual modules, summarized in Table 1. For most users, only three groups matter on a daily basis: the user TMB‐interface, the distributions, and the simulation and diagnostics tools. These are described below. The remaining modules are available for advanced use and described in vignette(“RTMB‐advanced”).

**TABLE 1:** Modules of the RTMB package and the functionality each provides. These modules can be accessed via the standard R help system (e.g., ?TMB‐interface).

Module	What it provides
TMB‐interface	The main user‐facing functions: MakeADFun(), getAll(), OBS(), REPORT(), ADREPORT(), sdreport(), checkConsistency()
Distributions, MVgauss	Standard and extended probability distributions (scalar and multivariate) that work transparently during fitting, simulation, and residual computation
Simulation, OSA‐residuals	Automatic simulation from the fitted model (obj$simulate()) and one‐step‐ahead quantile residuals (oneStepPredict())
Tape	Low‐level tape construction (MakeTape()) and control (TapeConfig(), DataEval(), GetTape())
ADvector, ADsparse, ADcomplex, ADmatrix	AD‐aware numeric, sparse, complex, and matrix types that look and behave like their ordinary R counterparts
ADjoint, ADoverload, Interpolation, ADintegrate, ADuniroot, ADapply, expAv	Advanced tools for custom derivatives, differentiable interpolation, integration, and root‐finding

### 2.2 User TMB ‐interface

For most users, RTMB::MakeADFun() is the only function they will ever need. The user passes an objective function and a list of starting parameter values to the function, and it returns a model object ready for optimization.

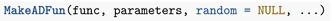

Unlike TMB::MakeADFun(), there are no data or DLL arguments. The objective function func is a plain R closure that captures any data it needs from its surrounding environment, and no pre‐compiled C++ library is required. All other arguments (…) behave identically to their TMB counterparts. The random argument is the key one for hierarchical models: any parameters listed there are treated as random effects and integrated out by the Laplace approximation rather than being directly optimized.

Internally, MakeADFun() builds three tapes (Kristensen et al. 2016): one for the user's objective function, one for its gradient, and one for its sparse Hessian. These tapes are used as building blocks for the Laplace approximation and its derivatives. This is exactly the same three‐tape machinery as in TMB; RTMB merely replaces the compiled C++ template with an R function.

Four further helper functions are available to be used inside the objective function:

- getAll() is a convenience function that unpacks one or more named lists directly into the current function's environment, so that data and parameters can be referred to by name, see a simple example below for arbitrary observations dat$Y and parameters p$mu and p$sigma:

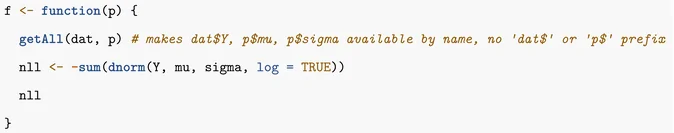

- OBS(Y): marks Y as an observation. This one‐line addition is what enables automatic simulation (obj$simulate()) and one‐step‐ahead residuals (oneStepPredict(obj)). Without it, RTMB does not know which quantities in the function are data and which are parameters, so it cannot substitute random draws during simulation.
- REPORT(x): saves x so it can be retrieved after fitting via obj$report(), without contributing to the uncertainty propagation.
- ADREPORT(x): flags x as a derived quantity for which sdreport() should compute standard errors automatically via the delta method (i.e., error propagation through the parameter uncertainty).

The model object returned by RTMB::MakeADFun() is structurally identical to the one returned by TMB::MakeADFun(), so every downstream tool works without modification: nlminb() (or any other optimizer) for fitting, sdreport() for uncertainty quantification, oneStepPredict() for residual diagnostics (simplified call in RTMB), and checkConsistency() for simulation‐based self‐checks. The latter function checks that the simulation code of random effects and data are consistent with the implemented negative log‐likelihood function. It also provides an estimate of parameter bias caused by the Laplace approximation.

### 2.3 Distribution Overloading, Simulation, and OSA Residuals

RTMB supports a wide range of probability distributions, including all the standard ones from R's stats package (e.g., dnorm(), dbinom(), dpois(), dgamma()), as well as extended distributions common in applied ecology and epidemiology (e.g., dtweedie(), dcompois()), and multivariate Gaussian (e.g., dmvnorm(), dgmrf(), dautoreg()). The R package RTMBdist (Fischer 2026) provides additional non‐standard distributions.

What makes these distributions special in RTMB is that a single call such as dnorm(Y, mu, sigma, log = TRUE) serves three distinct roles depending on the context in which it is evaluated, without any changes to the user's code:

1. **During fitting**: evaluates the log‐density and records the operation on the AD tape so that gradients can be computed.
2. **During simulation** (when obj$simulate() is called): draws a random value from the distribution and stores it in Y. This is why OBS(Y) is needed; it tells RTMB that Y is the quantity to be replaced by a simulated draw.
3. **During residual computation** (when oneStepPredict() is called): evaluates the one‐step‐ahead conditional cumulative distribution function, which is used to convert model predictions into quantile residuals for model checking (Thygesen et al. 2017).

This three‐in‐one behavior means that writing the likelihood once is sufficient for fitting, simulation‐based validation, and residual diagnostics, leading to a significant reduction in code compared to TMB, where simulation requires separate SIMULATE blocks, and OSA residuals need a data indicator.

One practical note: during simulation, RTMB generates random draws in the order they are encountered in the objective function. This means the sequence in which densities are evaluated matters for simulation (though not for fitting). In most models written in a natural top‐to‐bottom style, this is automatic, but it is worth being aware of for models with complex dependency structures.

## 3 An Implementation Example in RTMB and Comparison With TMB: The Theta‐Logistic Population Model

Fitting models in RTMB always follows these three general steps:

1. **Model definition**: R closure containing the full model and returning the objective function.
2. **Data and initial parameters**: load and define a data object and an initial parameter values object. Initial parameters should be gathered in a list.
3. **Model object and optimization**: call the MakeADFun() function to build the TMB object including notably the objective function, the initial parameter list, and the vector of random effect names (if any) as arguments; then optimize using the optimizer of choice (e.g., nlminb()).

The following additional steps might also be relevant:

4 **OSA residuals estimation**: run oneStepPredict() to compute the OSA residuals.
5 **Estimate parameter uncertainty**: run sdreport() to calculate the standard deviation of all model parameters and variables specified in the objective function with ADREPORT().
6 **Extract reported quantities**: if REPORT() was used in the objective function, these can be extracted via obj$report().
7 **Check the implementation** via simulation: if the model is well implemented, running checkConsistency() should return “Simulation appears to be correct”.
8 **Simulate** from the fitted model: if a simulation study is of interest, random effects, reported values, and data can be simulated using obj$simulate().

The RTMB workflow is illustrated using the theta‐logistic state‐space model (Wang 2007; Pedersen et al. 2011), a canonical TMB example that estimates population growth rates and carrying capacity from abundance time series (here slightly modified for convenience). The model extends logistic growth with a shape parameter θ controlling density dependence, and separates process variability from observation error. We also provide a side‐by‐side comparison with the equivalent TMB implementation where it differs; readers new to RTMB can instead start with vignette(“RTMB‐introduction”).

### 3.1 Model Specification

The theta‐logistic state‐space model relates a latent log‐abundance process $X_{t}$ to observations $Y_{t}$:

$$X_{t}=X_{t-1}+r_{0}\left[1-{\left(\frac{e^{X_{t-1}}}{K}\right)}^{\theta }\right]+\eta_{t},\;\;\eta_{t}∼N\left(0,Q\right),\;\;t=2,\ldots ,T$$

$$Y_{t}=X_{t}+\varepsilon_{t},\;\;\varepsilon_{t}∼N\left(0,R\right),\;\;t=1,\ldots ,T$$

Parameters are estimated on the log scale, that is, $\log r_{0}$, logθ, logK, logQ, logR, to avoid instability as some of them might approach zero. The latent states $X_{t}$ are random effects integrated out via the Laplace approximation. For convenience, we define $X_{1}=3$.

### 3.2 Model Code

The first part of the TMB and RTMB workflow (steps 1–4) is shown in Figure 1 with TMB on the left (the C++ template thetalog.cpp followed by its controlling R script) and the equivalent RTMB implementation as a single R file on the right. Inline comments and arrows highlight the points at which the two frameworks differ; everything else is mechanically equivalent. The side‐by‐side comparison reveals several structural differences with practical consequences:

- **Elimination of the C++ file** (step 1). TMB requires a .cpp file in addition to the controlling R script. RTMB needs only R, reducing the total file count from two to one and the total lines of code by roughly 37% for this particular example.
- **Syntax difference** (step 1). TMB C++ requires every intermediate value to use the Type template parameter. In RTMB, standard R syntax is used, and the type is deduced at runtime depending on the context. Note that exponentiation also differs syntactically: pow(x, theta) in C++ is simply x^theta in R.
- **Indexing convention** (step 1). TMB uses zero‐based indexing (inherited from C++), so the first element is X[0] and loops run from i = 0. RTMB follows R's convention, where the first element is X[1] and loops start at i = 1.
- **Automatic simulation** (step 1). TMB supports obj$simulate(), but only if the user adds a matching SIMULATE block beside every density call in the C++ template. In RTMB, the same dnorm() call is automatically reinterpreted in simulation mode.
- **No compilation step** (step 2). The compile() and dyn.load() calls in TMB add 5–60 s of latency per model change. RTMB retapes instantly when the R function or parameters change.
- **No data argument in MakeADFun()** (step 3). RTMB::MakeADFun() does not require a data argument contrarily to TMB::MakeADFun(). If needed, for example when creating a wrapper function fitting the same model to different data sets, one can add a data argument to the function in step 1 (e.g., function(p, data){}) as described in the section “Using an explicit data argument” in vignette(“RTMB‐introduction”).
- **Simplified code for residuals** (steps 1 and 4). In TMB, to compute OSA residuals, the C++ code needs to define a data indicator (e.g., DATA_VECTOR_INDICATOR(keep,Y)) and to include it in the negative log‐likelihood (step 1). Additional arguments are also needed when running oneStepPredict() (step 4).

**FIGURE 1:**
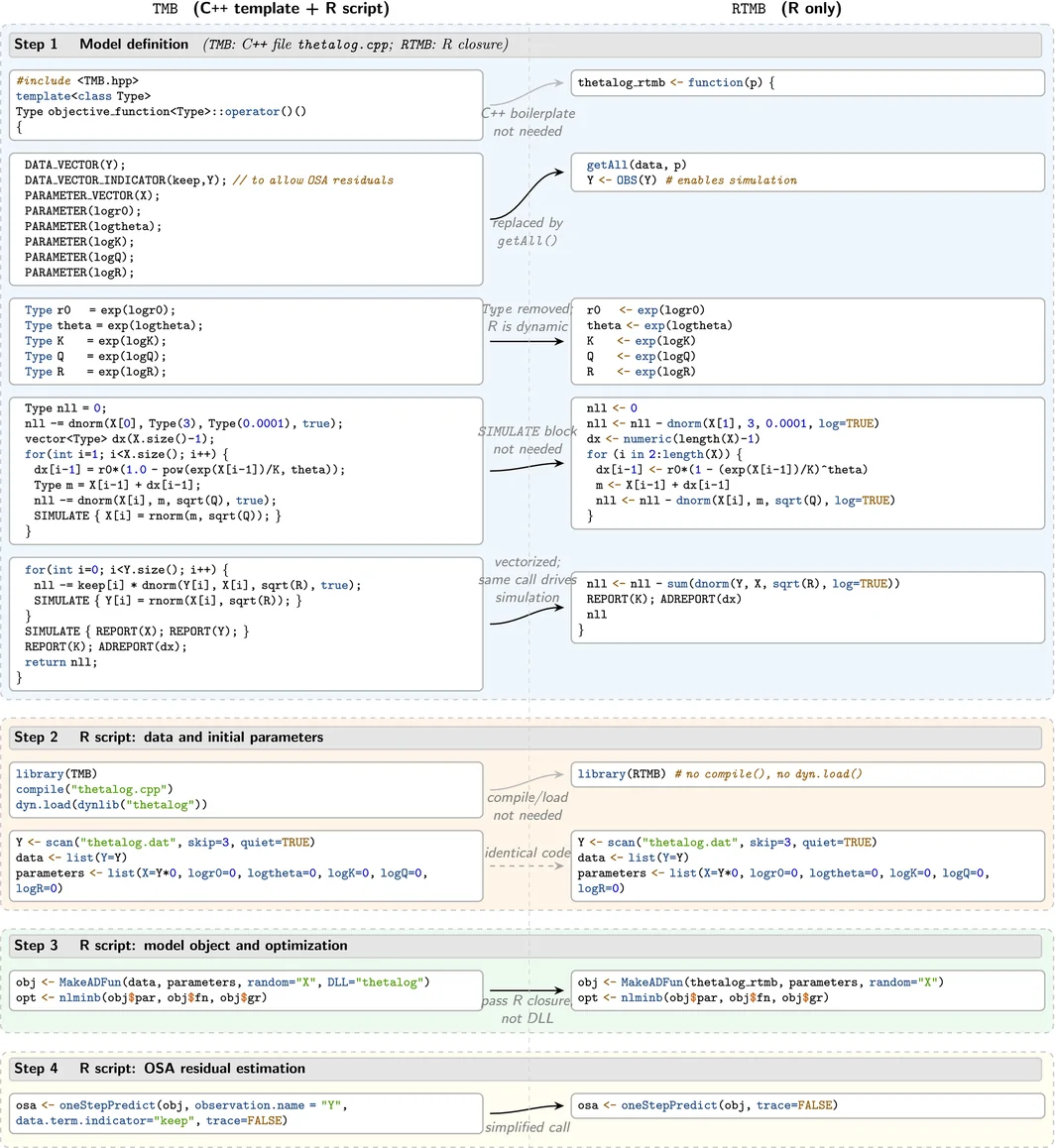
Annotated sub‐box comparison of TMB and RTMB for the theta‐logistic model. Related lines are grouped into sub‐boxes; arrows show how each TMB construct maps to its RTMB equivalent. Solid black arrows = structural transformation; dashed gray arrows = identical or near‐identical code; solid gray arrows = C++ constructs absent in RTMB.

After steps 1–4, the code is the same for both RTMB and TMB and is described below. Both frameworks have numerical equivalence and produce identical parameter estimates and standard errors. Results are shown in Figure 2, and the TMB and RTMB scripts are available in Supporting Information.

**FIGURE 2:**
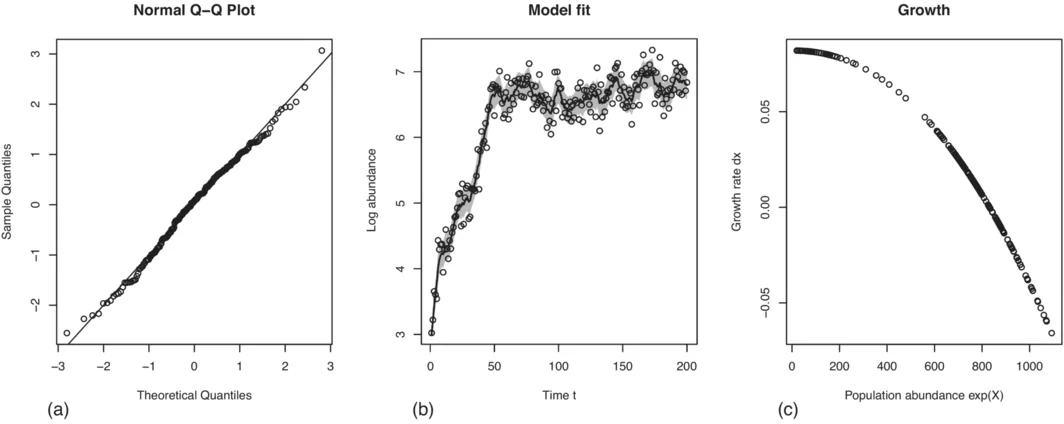
Residual plot showing normality (a), model fit to the observations (b), and growth rate against abundance (c). In (b), dots are the observations (Y), the line is the random effect (X), and the gray area is the 95% confidence interval around X.

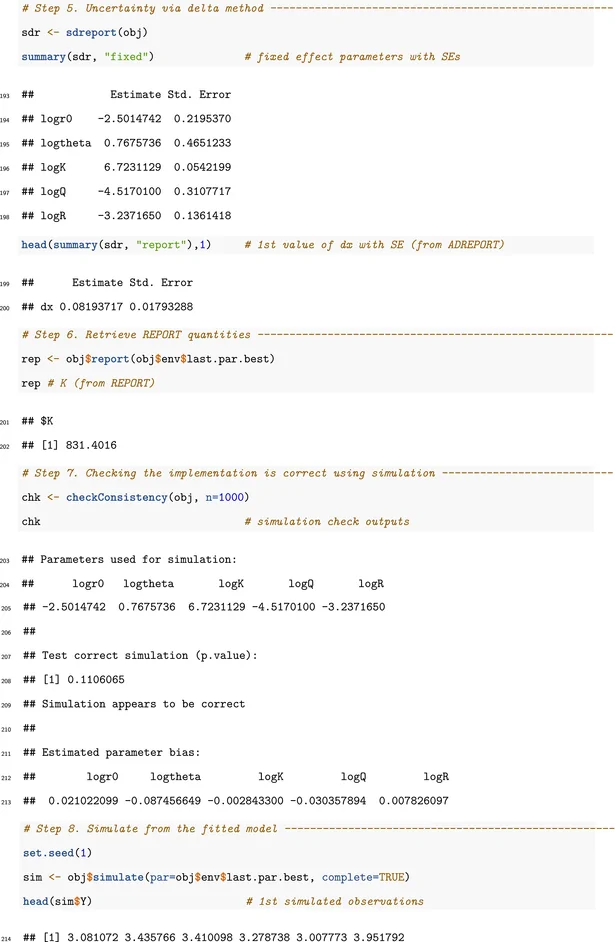

### 3.3 Runtime Comparison

Table 2 compares the runtime difference between TMB and RTMB for the relevant processes. These were obtained on one machine using the system.time() function.

**TABLE 2:** Runtime (in second) for the relevant TMB and RTMB processes for the theta‐logistic example.

Process	Code	TMB	RTMB
Compilation	compile(“thetalog.cpp”)	36.29	NA
Tape generation	obj <‐ MakeADFun(…)	0	0.02
Optimization	opt <‐ nlminb(obj$par, obj$fn, obj$gr)	0.22	0.22
Total		36.52	0.24

While the above example is not complex, it shows the trend that would be observed with most models. The optimization takes the same time on both platforms and what differs is the compiling time (absent in RTMB) and the tape generation that can take longer in RTMB. It would be possible to reduce the tape generation time in RTMB by making the code more efficient, for example, by removing the for loops and using vectorization.

For most cases, runtime difference is not large enough to be considered pertinent. However, runtime is particularly relevant in simulation‐estimation studies that require many iterations of tape generation + optimization. Compiling time is irrelevant here as compilation is only run once. In this case, user awareness is needed to reduce tape generation time in RTMB.

## 4 Discussion

By removing the C++ barrier while preserving TMB's performance, RTMB lowers the cost of developing and refining hierarchical latent variable models.

### 4.1 Practical Advantages for Education and Research

RTMB's plain‐R interface offers concrete advantages for both teaching and research. Models require no compiled binaries, making them portable across platforms and easy to share. Standard R debugging tools (browser(), debug(), print()) work directly inside the objective function, in contrast to debugging a C++ template. An instructor can move from a plain likelihood to a full hierarchical model fitted via the Laplace approximation (including simulation and consistency checks) entirely within R and without introducing a second language. In a research setting, the same workflow eliminates the C++ compilation pipeline and the obj$simulate() / checkConsistency() machinery provides a ready framework for parametric bootstrap and posterior predictive checks.

Because RTMB and TMB produce structurally identical MakeADFun() objects, RTMB is compatible with the broader TMB ecosystem, including tmbstan (Monnahan and Kristensen 2018), glmmTMB, sdmTMB, and OSA residual diagnostics. Existing TMB models can be converted by replacing the C++ template with an R function while leaving the downstream pipeline unchanged.

### 4.2 RTMB in the Landscape of Latent Variable Software

RTMB occupies a particular niche in the R‐based statistical‐modeling landscape. Three natural points of comparison are Stan, INLA, and NIMBLE.

Stan (Carpenter et al. 2017) targets full Bayesian inference via Hamiltonian Monte Carlo, using its own modeling language and requiring a compilation step. It incurs substantially longer runtimes than RTMB's Laplace approximation for models with many random effects. tmbstan bridges the two approaches by applying a Stan sampler to a TMB object when full posteriors are needed. The combination of tmbstan with RTMB therefore allows fitting Stan models without a compilation step.

INLA (Rue et al. 2009; Lindgren and Rue 2015) offers computationally efficient approximate Bayesian inference for latent Gaussian models but does not provide a general interface for arbitrary user‐defined likelihoods. NIMBLE (de Valpine et al. 2017) provides flexible model specification via a domain‐specific language but requires a compilation step.

The distinctive offering of RTMB is therefore the combination of (i) a general‐purpose marginal likelihood, (ii) full automatic differentiation in pure R without a separate modeling language and a compilation step, and (iii) competitive runtime performance relative to MCMC‐based alternatives.

### 4.3 Limitations

Some advanced TMB features, notably OpenMP parallelism and special functions compiled with forward mode AD, are not fully supported or require workarounds.

While RTMB offers a strong advantage for interactive, iterative model building, TMB's explicit type declarations (e.g., Type, vector<Type>) enforce a stricter, compiler‐checked model definition.

Unlike TMB's pre‐compiled C++, RTMB builds its computational tape using plain R, which introduces a modest upfront time cost. As mentioned in Section 3.3, models with explicit R for loops over long time series can be slow in the tape generation step (i.e., when running MakeADFun()) because each loop iteration is traced individually. Vectorization, either explicitly or via the Vectorize() function, can mitigate this but requires user awareness. Likewise, tape generation can be slow if R objects are copied on modification.

Conditional code paths that depend on parameters (e.g., if (x > 0)) are non‐differentiable and require TapeConfig(comparison = “tape”) or ADoverload(“if”) to be handled correctly.

RTMB differentiates a function by promoting its numeric inputs to a special advector type that records every arithmetic operation performed on it, building a tape that can later be replayed to compute derivatives. A function is AD‐compatible if it is built entirely from R‐level arithmetic and math operations that RTMB overloads for advector (e.g., +, *, exp(), sum(), indexing). Functions from external R packages that instead call compiled C, C++, or Fortran code internally (as many numerical routines do) generally are not AD‐compatible, since compiled code expects ordinary double values rather than advector objects; such functions must be extended with derivative information using ADjoint().

### 4.4 Concluding Remarks

RTMB is under active development. Online documentation, including vignettes and worked examples, is available at https://kaskr.r‐universe.dev/RTMB and will continue to expand alongside the package. Since its initial release in 2023, RTMB has seen steady uptake. It is already serving as the foundation for a growing number of dependent packages across ecology, fisheries, and epidemiology.

In summary, RTMB brings TMB's automatic differentiation and Laplace approximation to native R, with negligible loss of performance, making latent variable modeling accessible to a broader community of researchers and students.

## Author Contributions

**Kasper Kristensen:** conceptualization (lead), formal analysis (lead), investigation (lead), methodology (lead), software (lead), validation (lead), writing – original draft (supporting), writing – review and editing (equal). **Anders Nielsen:** conceptualization (supporting), investigation (supporting), methodology (supporting), validation (supporting), writing – review and editing (equal). **Christoffer Moesgaard Albertsen:** conceptualization (supporting), investigation (supporting), methodology (supporting), validation (supporting), writing – review and editing (equal). **Casper Willestofte Berg:** conceptualization (supporting), investigation (supporting), methodology (supporting), validation (supporting), writing – review and editing (equal). **Vanessa Trijoulet:** conceptualization (supporting), investigation (supporting), methodology (supporting), validation (supporting), writing – original draft (lead), writing – review and editing (equal).

## Funding

This work and open access publishing were facilitated by the section for Marine Living Resources at the National Institute of Aquatic Resources, Technical University of Denmark.

## Conflicts of Interest

The authors declare no conflicts of interest.

## Supporting information


Data S1.

## Data Availability

All package code is available at https://github.com/kaskr/RTMB and documentation at https://kaskr.r‐universe.dev/RTMB.
